# Mindfulness-Based Intervention for Treatment of Anxiety Disorders During the Postpartum Period: A 4-Week Proof-of-Concept Randomized Controlled Trial Protocol

**DOI:** 10.3390/brainsci16010088

**Published:** 2026-01-13

**Authors:** Zoryana Babiy, Benicio N. Frey, Randi E. McCabe, Peter J. Bieling, Luciano Minuzzi, Christina Puccinelli, Sheryl M. Green

**Affiliations:** 1 Department of Psychology, Neuroscience & Behaviour, McMaster University, Hamilton, ON L8S 4L8, Canada; 2 Department of Psychiatry and Behavioural Neurosciences, McMaster University, Hamilton, ON L8S 4L8, Canada; 3 Women’s Health Concerns Clinic, St. Joseph’s Healthcare Hamilton, Hamilton, ON L9C 0E3, Canada

**Keywords:** mindfulness, mindfulness-based intervention, postpartum anxiety, anxiety, emotion regulation, interoception, fMRI, functional connectivity

## Abstract

**Background/Objectives**: Anxiety disorders (ADs) affect up to 20% of mothers in the postpartum period, characterized by psychological symptoms (e.g., emotion dysregulation; ER) and physical symptoms (e.g., disrupted bodily awareness). Although Cognitive Behavioural Therapy effectively reduces anxiety and mood symptoms, it shows limited efficacy in addressing ER difficulties and rarely targets interoceptive dysfunction—both common in postpartum ADs. This study evaluates the effectiveness of a brief mindfulness-based intervention in improving anxiety, ER, and interoception in mothers with postpartum ADs. A secondary aim is to examine changes in brain connectivity associated with these domains. **Methods**: This protocol describes a proof-of-concept randomized controlled trial involving 50 postpartum mothers with ADs. Participants will be randomized to receive either a 4-week mindfulness intervention plus treatment-as-usual (TAU) or TAU alone. Participants in the mindfulness + TAU group will complete a virtual 4-week group intervention adapted from Mindfulness-Based Cognitive Therapy. The TAU group will receive usual care for 4 weeks and then be offered the mindfulness intervention. Self-report measures of anxiety, ER, and interoception will be collected at baseline, post-intervention, and at a 3-month follow-up. Resting-state functional MRI will be conducted at baseline and post-intervention to assess functional connectivity changes. This trial has been registered on ClinicalTrials.gov (NCT07262801). **Results:** Improvements in anxiety, ER, and interoception are anticipated, along with decreased default mode network, and increased salience network connectivity post-intervention is hypothesized. **Conclusions**: This study will be the first to examine the combined psychological and neural effects of mindfulness in postpartum ADs, offering a potentially scalable mind–body treatment.

## 1. Introduction

### 1.1. Impact of Postpartum Anxiety Disorders

Anxiety disorders (ADs) affect approximately one in five mothers and birthing parents (hereafter referred to as “mothers/BPs”) during the postpartum period (birth up to one year following delivery) [1,2]. These disorders are associated with significant distress and impair a mother/BP’s ability to care for themselves and their infant [3] and have been linked to lower parenting competence [4], lower marital satisfaction [5], and a higher risk of substance use and suicide [6]. Mothers/BPs with ADs are also less likely to breastfeed [7] and more likely to experience difficulties in responsiveness and bonding [8]. These disruptions are associated with adverse child outcomes, including challenges with temperament (e.g., less easily soothed), emotion regulation (e.g., developing anxiety disorders), and behavioural problems in early childhood [9,10,11,12,13]. Despite the significant prevalence and impact of ADs during the postpartum period, their underlying mechanisms remain unclear, and effective nonpharmacological treatments that are scalable and durable are limited.

### 1.2. Emotion Dysregulation and Interoception: Emerging Targets

Increasing evidence points to emotion dysregulation as a critical underlying process in ADs, including those that occur during the postpartum period. Perinatal changes to the endocrine and hypothalamic–pituitary–adrenal (HPA) axis heighten vulnerability to emotional instability [14], and even in the absence of clinical levels of anxiety, perinatal individuals show greater emotional reactivity and mood fluctuations [15,16,17,18]. Maternal emotion dysregulation, in turn, has been linked to reduced supportive parenting, as well as infant emotion dysregulation and later adjustment problems [19,20,21]. Among those with clinical levels of postpartum anxiety and/or depression, lower positive affect, higher negative affect, and reduced emotional flexibility have been observed compared to healthy perinatal individuals [22]. Yet despite the clear link between emotion dysregulation and postpartum anxiety, emotion regulation is rarely addressed as a direct target in psychological interventions for ADs during the postpartum period.

Anxiety symptoms have also been linked to disrupted interoception—the ability to sense, interpret, and regulate internal bodily signals [23]. Most existing research has focused on cardiac interoception in non-perinatal populations, with no studies to date examining postpartum mothers/BPs with ADs [24]. For example, individuals with generalized anxiety disorder have shown altered heartbeat-evoked potentials, suggesting impaired cortical processing of interoceptive signals [25]. A recent meta-analysis also found that anxiety was associated with negative interpretations of bodily sensations, hypersensitivity to internal cues, and difficulty articulating emotional or physical states [26]. These findings position disrupted interoception as a potentially important yet understudied mechanism in postpartum anxiety, one that, like emotion regulation, may be valuable to target in future interventions.

### 1.3. Current Treatments for Postpartum Anxiety Disorders

Practice guidelines in Canada [27,28], the UK [29], and the USA [30] recommend non-pharmacological approaches as first-line treatments for perinatal anxiety. Cognitive behavioural therapy (CBT) is the most widely supported psychological treatment for ADs [31], though its effects on core mechanisms like emotion regulation and interoception remain underexplored. Existing evidence suggests that emotion dysregulation reduces CBT’s effectiveness in several anxiety populations [32,33], yet only one study has examined this in perinatal individuals. Agako et al. [34] found that mothers with high pre-treatment emotion dysregulation remained dysregulated post cognitive behavioural group therapy, suggesting a need for more targeted interventions. Similarly, although disrupted interoception is increasingly recognized as central to the development and maintenance of anxiety [24], its role in the treatment of ADs during the postpartum period has not been studied.

### 1.4. Mindfulness-Based Interventions

In light of the limitations of existing psychological interventions—namely, their lack of explicit focus on emotion dysregulation and disrupted interoception as core components of anxiety—mindfulness-based interventions (MBIs) have emerged as a compelling alternative. Mindfulness teaches individuals how to improve their relationship with their thoughts, emotions, and bodily sensations, addressing both psychological and physical symptoms [35,36]. MBIs show moderate effectiveness in reducing anxiety and improving mood in both clinical [37,38,39,40] and non-clinical populations [41,42,43], with emerging evidence suggesting that they are also associated with neurobiological changes in brain networks supporting emotion regulation (e.g., the salience network; SN) [44], self-awareness (the default mode network; DMN) [45], and executive function (the central executive network; CEN) [46].

The current literature on MBIs in perinatal populations has predominantly focused on pregnancy, where consistent improvements in anxiety and depressive symptoms have been reported (medium–large effect sizes) [47,48,49,50,51,52]. In contrast, postpartum studies are limited and often include mixed clinical and subclinical anxiety samples [53,54,55]. Critically, no study to date has targeted postpartum individuals with diagnosed ADs or examined associated neurobiological outcomes, highlighting a key gap and opportunity for intervention.

### 1.5. Current Study

Given the physical and psychological components of ADs and the promising effects of mindfulness intervention on anxiety, emotion regulation, and interoception in non-postpartum populations, we developed a mindfulness protocol for postpartum mothers/BPs with ADs. This proof-of-concept randomized controlled trial will examine the effects of a 4-week mindfulness-based group intervention compared to treatment as usual (TAU). The primary aim is to evaluate the intervention’s impact on AD symptoms using both subjective (self-reported anxiety, emotion regulation, and interoception) and objective (functional connectivity of brain networks associated with these processes) measures. We hypothesize that participants will show reductions in anxiety and improvements in emotion regulation and interoception. We also expect to observe changes in functional connectivity in relevant brain networks (e.g., the DMN and SN). To our knowledge, this will be the first study to assess the efficacy of a mindfulness protocol for postpartum mothers/BPs with ADs using a combination of self-report and neuroimaging outcomes.

## 2. Materials and Methods

### 2.1. Inclusion/Exclusion Criteria

Participants will be included in this trial if they meet the following criteria: (1) Mothers/BPs 18 years and older who are between 0 and 12 months postpartum; (2) principal diagnosis of an AD as per the Diagnostic Assessment Research Tool (DART) [56,57], with or without comorbid mood disorder; (3) no concurrent psychological treatment; (4) not taking psychoactive medication or (a) medications are stable in dose and type for at least 8 weeks prior to the study and (b) medications remain stable throughout the study; (5) fluent in English, minimal grade 8 reading level. Exclusion criteria are as follows: (1) Severe depression/suicidality requiring acute intervention; (2) presence of current psychotic symptoms or alcohol or substance use disorders; (3) suffering from claustrophobia; (4) metallic objects in the body (e.g., metal implants, pacemakers) or any other contraindication for brain MRI procedures (e.g., currently pregnant).

### 2.2. Sample Size

In keeping with the minimum of 30 participants required to estimate a parameter in pilot and proof-of-concept trials [58,59], and based on prior studies applying neuroimaging to evaluate the impact of a mindfulness-based group intervention in non-perinatal anxiety and mood populations [37,38,60,61,62,63,64], a minimum of 20 participants will be randomized to each arm. Accounting for an estimated 20% attrition, we will recruit 50 participants total (25 per arm).

### 2.3. Study Design and Procedure

**Figure 1:**
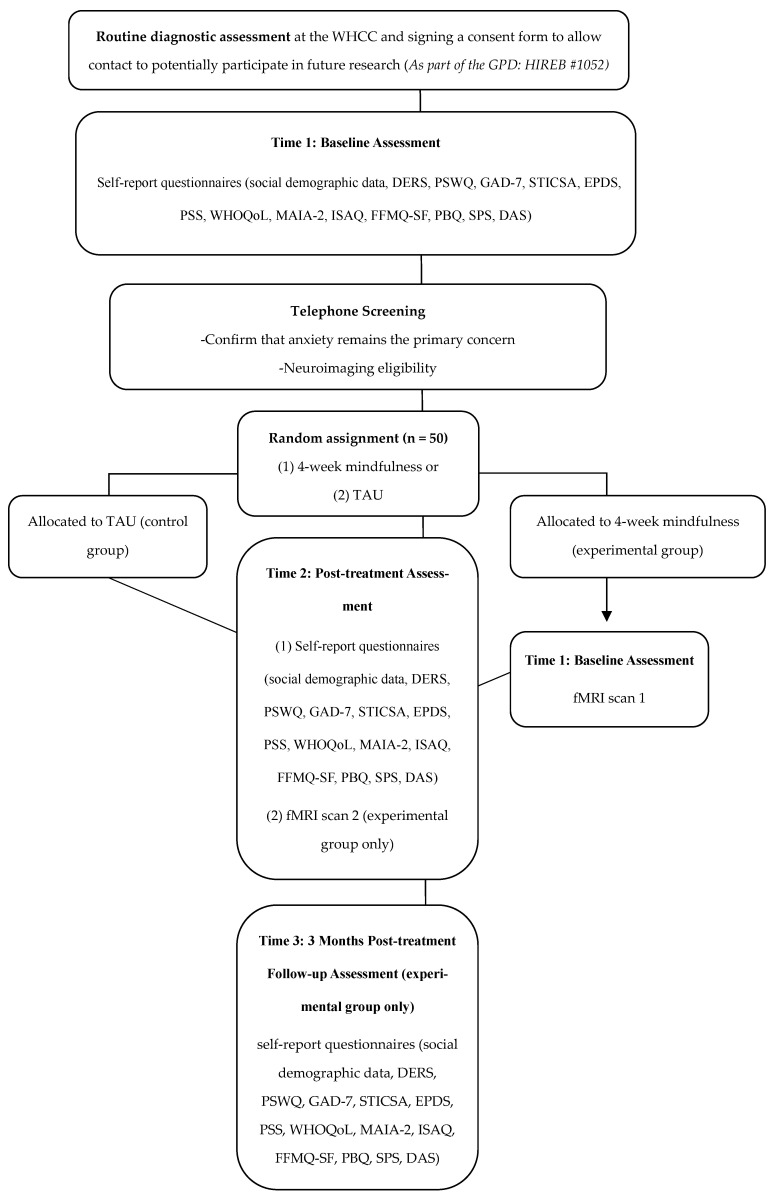
Flowchart of the study design. Green Prospective Database, GPD; Women’s Health Concerns Clinic, WHCC; DART, Diagnostic Assessment Research Tool; TAU, treatment as usual; DERS, Difficulties in Emotion Regulation Scale; PSWQ, Penn State Worry Questionnaire; GAD-7, Generalized Anxiety Disorder 7-Item Scale; STICSA, State-Trait Inventory for Cognitive and Somatic Anxiety; EPDS, Edinburgh Postnatal Depression Scale; PSS, Perceived Stress Scale; WHOQoL, World Health Organization Quality of Life Scale; MAIA-2, Multidimensional Assessment of Interoceptive Awareness 2; ISAQ, Interoceptive Sensitivity and Attention Questionnaire; FFMQ, Five Facet Mindfulness Questionnaire Short Form; PBQ, Postpartum Bonding Questionnaire; SPS, Social Provisions Scale; DAS, Dyadic Adjustment Scale; fMRI, functional magnetic resonance imaging.

This study is an investigator-initiated, single-centre, proof-of-concept randomized controlled trial. The study will be conducted within the Women’s Health Concerns Clinic (WHCC) and the Imaging Research Center (IRC), St. Joseph’s Healthcare Hamilton, Canada. As part of standard care within the WHCC, clinicians who believe a patient may benefit from psychological treatment for postpartum anxiety and who have agreed to be contacted for research (i.e., individuals who agreed to complete self-report demographic and clinical questionnaires as part of the Green Prospective Database; HiREB # 1052) may refer them to a group-based intervention. If they meet eligibility criteria based on their routine pre-treatment diagnostic assessment, a study staff member will contact them to confirm interest and finalize study eligibility (Figure 1).

Eligible participants will be contacted via telephone following their routine care diagnostic assessment by study staff to gauge interest in participation in the present pilot randomized controlled trial. If interested, participants will be sent a password-protected information and consent form via e-mail while on the phone with study staff. During the phone call, study staff will review the consent form with the participant, providing the opportunity to ask questions and clarify the risks and rules associated with Zoom. Following the informed consent process, participants will be asked to sign a copy of the consent form, using a digital signature, while on the phone with study staff, to ensure that it is the participant signing the form. Once signed, the participant will email the signed consent form back to the study staff, the study staff will add their digital signature to the consent form, and will return a copy of the signed consent form to the participant via email.

Eligible and consenting participants will be randomly assigned to either the experimental (4-week mindfulness-based group intervention) or the TAU control group. Participants will be randomized using block randomization, with a 1:1 allocation ratio, using a computer-generated assignment sequence prepared prior to the study. Participants randomized to the mindfulness-based intervention group will complete a battery of self-report questionnaires post-treatment (Time 2) and 3 months post-treatment (Time 3). Questionnaires completed at Time 2 and Time 3 will be the same as those completed at baseline (Time 1) as part of the Green Prospective Database. Participants randomized to the TAU control group will complete the same battery of self-report questionnaires at Time 2. All questionnaires will be completed via Research Electronic Data Capture (REDCap). Participants randomized to the experimental group will also participate in an imaging session at the IRC at Time 1 and Time 2.

Participants randomized to the mindfulness-based intervention group will receive a $20 e-gift card for completing baseline questionnaires, post-treatment questionnaires, and another $20 after completing the 3-month follow-up questionnaires. They will also receive a $30 e-gift for participating in fMRI scans at baseline and post-treatment (for a total maximum of $120). Participants randomized to the TAU control group will receive a $20 e-gift card for completing baseline questionnaires and $20 after completing questionnaires following the 4-week TAU period (for a total maximum compensation of $40).

In addition to receiving financial compensation and the intervention being delivered entirely online, other attrition mitigation strategies have been put in place. Study staff will maintain regular contact via email and phone to remind participants about sessions and questionnaire-completion. Informal childcare support will be provided during MRI appointments, where a trained research assistant will be present while the participant completes the scan.

### 2.4. Risk Assessment

Screening for severe depression and suicidality is conducted as part of routine intake at the Women’s Health Concerns Clinic, prior to participants being screened for our study. These individuals would be identified as requiring acute intervention and excluded from the study’s recruitment and referred to appropriate clinical services. However, if imminent risk is identified during completion of the risk module of the Diagnostic Assessment Research Tool (DART), as part of the screening for the study, a safety planning process will be initiated by the assessor with the participant via Zoom. If a sufficient safety plan cannot be established, standard clinical escalation procedures will be implemented in accordance with the hospital’s protocols. Risk will be monitored throughout study participation, and all participants with a history of suicidality or current risk will be provided with crisis resources, including the Crisis Outreach and Support Team (COAST).

### 2.5. Study Arms

The 4-week mindfulness-based group intervention involves participating in a 4-week group that meets once a week, for 2 h. The session will be delivered through Zoom. Sessions will be delivered by a senior-level Ph.D. student in clinical psychology (ZB) and a clinical psychologist (SMG, CP). Each session will consist of psychoeducation about anxiety during the perinatal period and mindfulness practices designed to foster a state of mindfulness and improve anxiety symptoms. To encourage daily practice, links to guided meditation (i.e., SoundCloud links) will be included in the mindfulness protocol shared with participants. The intervention protocol was adapted from existing evidence-based protocols, including the Mindfulness-based Cognitive Therapy for depression protocol [65], as well as other perinatal-specific protocols [25,50,52,55]. The content provided in each session is detailed in Table 1.

Intervention fidelity will be monitored using a session-level adherence checklist. A trained research assistant will be present for the full duration of each group session and will complete the checklist following each session to document adherence to core intervention components (e.g., psychoeducation content, mindfulness practices, and session structure). These procedures are intended to ensure the consistency of intervention delivery across sessions and groups.

The minimum attendance requirement is attending 3/4 sessions to be included in the primary analysis. In instances where a participant cannot attend a session, an individual make-up session will be delivered by one of the group facilitators. In addition to tracking attendance, recording of any technical disruptions related to virtual delivery will also be recorded.

Participants in the TAU control group will engage in TAU for 4 weeks (waitlist period), which entails continuing to meet with their healthcare practitioners on an as-needed basis. This includes meeting with their psychiatry team, attending regular evaluations within the hospital, and/or meeting with other allied health professionals such as social workers, nurses, or counsellors. The one restriction that is placed on the 4-week waitlist period is not engaging in any formal, structured psychotherapy (e.g., CB, DBT, or other mindfulness-based interventions). After the 4-week TAU period, participants will be given the option to complete the 4-week mindfulness intervention.

### 2.6. Neuroimaging Visits

During each imaging session at the IRC, participants will review an information sheet along with their completed screener with the imaging technician. Each imaging session will last approximately 20 min, during which participants will be instructed to keep their eyes open while focusing on a fixation cross. As research scans are not intended for diagnosis, if an abnormality is seen on an MRI scan, no formal report will be issued, and participants will be advised to consult with their family doctor/general practitioner.

The attained images will later be compared to healthy control fMRI data from the Canadian Biomarker Integration Network in Depression (CAN-BIND), a standardized research platform that collects comprehensive data from both healthy and clinical samples. CAN-BIND data are comparable in terms of core MRI acquisition protocols; however, as historical control data, these comparisons may be influenced by unmeasured factors such as differences in resting-state instructions, time-of-day effects, and residual demographic or contextual mismatches, and are therefore interpreted as exploratory.

### 2.7. Participation Time Commitment

Total participation time for the experimental group is approximately 13–15 h across the 4-week intervention and follow-up period. This includes four 2 h virtual group sessions (8 h), completion of self-report questionnaires at three time points (piloting indicated that Time 1 requires approximately 15–20 min, as many measures are already part of the GPD [see page 5], and Time 2 and Time 3 each require less than 30 min; ~1–1.5 h total), two MRI sessions of approximately 20 min each (approximately 1 h including preparation), and strongly encouraged home mindfulness practice (recommended 10–20 min per day, flexible in timing). Participants in the TAU group complete approximately 1–1.5 h of questionnaire-related activities across two time points. As indicated, the participants will be compensated for all questionnaire and scan completions. Compensation is structured to acknowledge time and inconvenience rather than to function as an inducement. The compensation rates are consistent with those used in local perinatal mental health and neuroimaging research, including those in our research group, and were approved by the institutional Research Ethics Board.

### 2.8. Primary Outcomes

State–Trait Inventory for Cognitive and Somatic Anxiety (STICSA): The STICSA is a 21-item self-report scale that assesses cognitive and somatic components of anxiety [66]. The STICSA comprises a state scale that measures individuals’ current anxiety symptoms and a trait scale that assesses one’s proneness to anxiety, more generally. In the present study, only the trait scale of the STICSA will be used. Items are scored on a 4-point scale ranging from 1 (‘not at all’) to 4 (‘very much so’). The STICSA has demonstrated excellent validity and reliability [67]. A cut-off score of 43 or higher on the STICSA has been suggested for detecting a probable anxiety disorder [68]. The STICSA has been validated for use in clinical samples of adults with anxiety disorders [67]. Although not explicitly validated in perinatal samples, it has been used in treatment studies with perinatal populations [69,70].

### 2.9. Secondary Outcomes

Generalized Anxiety Disorder 7-Item Scale (GAD-7): The GAD-7 is a 7-item self-report measure that assesses anxiety symptom severity over the previous two-week period [71]. Items are measured on a 4-point scale ranging from 0 (‘not at all’) to 3 (‘nearly every day’). A cut-off score of 10 or higher with a sensitivity of 89% and specificity of 82% has been suggested for detecting a probable diagnosis of Generalized Anxiety Disorder [71]. The GAD-7 has also been validated for use in perinatal samples [72].

Penn State Worry Questionnaire (PSWQ): The PSWQ is a 16-item self-report measure that assesses the tendency to worry [73]. Items are scored on a 5-point scale ranging from 1 (‘not typical at all’) to 5 (‘very typical’), with higher scores reflecting greater pathological worry. A cut-off score of 62 or higher has been suggested for determining a probable GAD diagnosis [74]. The PSWQ has demonstrated excellent internal consistency and validity across various populations and has been validated for use in perinatal samples [75].

Difficulties in Emotion Regulation Scale (DERS): The DERS is a 36-item self-report scale designed to assess difficulties with emotion regulation [76]. The DERS contains six subscales that measure the following: non-acceptance goals, impulse, awareness, strategies, and clarity. Items are rated on a 5-point Likert scale, ranging from 1 (almost never, 0–10%) to 5 (almost always, 91–100%), with higher scores reflecting greater difficulties regulating emotions. The DERS has demonstrated good internal consistency (α = 0.86) and test–retest reliability (0.74) and has been validated for use in perinatal samples [77].

Multidimensional Assessment of Interoceptive Awareness 2 (MAIA-2): The MAIA-2 is a 37-item self-report scale that assesses multiple dimensions of interoception (i.e., awareness of bodily sensations) [78]. Items are scored on a 6-point scale ranging from 0 (never) to 5 (always), with higher scores equating to more awareness of bodily sensation. The MAIA-2 has 8 subscales assessing dimension of noticing, not-distracting, not-worrying, attention regulation, emotional awareness, self-regulation, body listening, and trust. The MAIA-2 has been validated for use in a variety of clinical and non-clinical samples [79,80], although not explicitly in perinatal samples. Although the MAIA-2 has not been explicitly validated in perinatal samples, it is included here as a theoretically relevant measure of interoceptive awareness; findings will be interpreted cautiously, and formal psychometric evaluation in postpartum populations is planned for a future large-scale trial.

Interoceptive Sensitivity and Attention Questionnaire (ISAQ): The ISAQ is a 17-item self-report measure assessing sensitivity and attention to interoceptive signals [81]. Items are scored on a 5-point scale ranging from 1 (strongly disagree) to 5 (strongly agree), with higher scores indicating greater interoceptive sensibility. The ISAQ has 3 subscales assessing sensitivity to neutral bodily sensations, attention to unpleasant bodily sensations, and difficulty disengaging from unpleasant bodily sensations. The ISAQ has been validated for use in a variety of clinical and non-clinical samples, although not explicitly in perinatal samples [81]. Given the absence of explicit validation in perinatal samples, ISAQ findings will be interpreted cautiously, and future studies will examine its psychometric performance and construct validity in postpartum populations.

Five-Facet Mindfulness Questionnaire Short Form (FFMQ-SF): The FFMQ-SF is a 24-item self-report scale that assesses mindfulness [82]. Items are scored on a 5-point scale ranging from 1 (never or very rarely true) to 5 (very often or always true), with higher scores indicating greater mindfulness. The FFMQ-SF assesses five aspects of mindfulness, including observing, describing, acting with awareness, non-judging, and non-reactivity. The FFMQ-SF has been validated for use in a variety of clinical and non-clinical samples, although not explicitly in perinatal samples [82,83].

Edinburgh Postnatal Depression Scale (EPDS): The EPDS is a 10-item self-report measure that assesses symptoms of depression during the perinatal period [84]. Items are scored on a 4-point scale ranging from 0 to 3, with higher scores reflecting greater depressive symptomatology. The EPDS has demonstrated good sensitivity and specificity for a diagnosis of Major Depressive Disorder (MDD). A cut-off score of 13 or higher has been suggested for detecting a probable diagnosis of MDD during the postpartum period [85]. Additionally, the EPDS has also been used to assess postpartum anxiety, with 3 of the 10 included questions specifically probing into anxiety symptoms (EPDS-3A) [86].

Perceived Stress Scale (PSS): The PSS is the most widely used psychological instrument for measuring the perception of stress [87]. The PSS is a 10-item self-report measure designed to assess the degree to which situations in one’s life are appraised as stressful [88]. Items are scored on a 5-point scale ranging from 0 (‘never) to 4 (‘fairly often’) with higher scores reflecting greater perceived stress. The PSS has demonstrated good psychometric properties [87].

Social Provisions Scale (SPS): The SPS is a 24-item self-report scale that assesses perceived social support received within social relationships [89]. Items are scored on a 4-point scale ranging from 1 (strongly disagree) to 4 (strongly agree), with higher scores indicating higher levels of perceived social support [89]. The SPS has 6 subscales assessing dimensions of attachment, social integration, reassurance of worth, guidance, nurturance, and reliance. The reliability of the subscales is adequate (alpha ranging from 0.65 to 0.76).

Postpartum Bonding Questionnaire (PBQ): The PBQ is a 25-item self-report measure that assesses maternal–infant relations [90]. Items are scored on a 6-point scale ranging from 0 to 5 [90]. The PBQ has 4 subscales, including bonding, rejection, and anger towards the infant, infant-focused anxiety, and incipient abuse. The PBQ has good sensitivity, particularly for the bonding subscale (0.82) and the rejection and anger towards the infant subscale (0.88).

The Dyadic Adjustment Scale (DAS): The DAS is a 32-item scale used to measure an individual’s feelings about their relationship with an intimate partner [91]. The items are scored on 2-, 5-, 6-, or 7-point scales, with higher scores indicating greater relationship satisfaction. The DAS contains four subscales, including dyadic consensus, dyadic satisfaction, dyadic cohesion, and affective expression. The DAS has good internal consistency (alpha = 0.80).

World Health Organization Quality of Life Scale (WHOQoL): The WHOQoL-BREF is a 26-item self-report measure that assesses quality of life across four domains, including physical health, psychological health, social relationships, and environment [92]. Items are scored on a 5-point Likert scale ranging from 1 to 5, with higher scores indicating greater quality of life. The WHOQoL-BREF has demonstrated good discriminant validity, content validity, internal consistency, and test–retest reliability [92].

### 2.10. Exploratory Outcome

Resting-state Functional Connectivity: MRI data will be acquired using a 3T Discovery MR750 scanner (manufacturer: GE HealthCare; Chicago, IL, USA). Experimental group participants will undergo a 10 min anatomical MRI, followed by a 10 min resting-state fMRI during which they will be instructed to fixate on a cross. Changes in resting-state functional connectivity within the DMN and SN will be evaluated to examine whether improvements in self-reported anxiety, emotion regulation, and interoception are associated with neurobiological changes.

### 2.11. Data Management

Data collection and storage for this study will consist of digital files. Participant questionnaires will be completed and electronically managed using REDCap. After obtaining informed consent, participants will be assigned a de-identified code, and only this code will be used to identify participants within REDCap. A list linking participant codes and names will be stored separately from all other documents on a separate, password protected excel file located on a secure drive, to ensure participant confidentiality.

The neuroimaging data will be stored separately in a password-protected drive and access will be limited to individuals directly involved with the study. Only the de-identified code will be used to identify participants within the password-protected drive.

### 2.12. Data Analysis

To determine condition differences on the self-report clinical outcomes, a series of repeated measures analysis of variance (ANOVAs) will be used. A two (experimental group vs. TAU group) by two (Time 1 vs. Time 2) repeated measures ANOVA will be used to examine differences. Further, in order to determine if benefits are maintained long-term, a within-subject repeated measures ANOVA will be conducted for the experimental group across three testing times (Time 1, Time 2, Time 2). The primary outcome (STICSA) will be evaluated at α = 0.05. All other self-report measures are secondary, pre-specified a priori, and Bonferroni correction will be applied to account for multiple comparisons where appropriate. Given the proof-of-concept nature of the study, findings from secondary outcomes will be interpreted cautiously. Partial eta-squared (η^2^) will be reported for ANOVA effects, and Cohen’s d with 95% confidence intervals will be calculated to inform future sample size calculations. Analyses will follow an intent-to-treat (ITT) approach, with missing data handled using appropriate methods (e.g., multiple imputation where assumptions are met). All analyses will be performed with IBM SPSS Statistics 23 [93].

Preprocessing of fMRI data will be conducted using a combination of SPM12 (http://www.fil.ion.ucl.ac.uk/spm/software/spm12; accessed on 22 November 2025), FSL (https://fsl.fmrib.ox.ac.uk/fsl/fslwiki/; accessed on 22 November 2025), and custom MATLAB (version R2025b) scripts. Preprocessing will include motion correction, coregistration to the structural image, spatial normalization, nuisance regression (motion parameters and white matter/cerebrospinal fluid signals), and temporal band-pass filtering. ROI-to-ROI functional connectivity analysis will be performed using SPM to evaluate changes in connectivity within the DMN and SN. A priori-defined regions of interest (ROIs) for the DMN include the medial prefrontal cortex and posterior cingulate cortex, and for the SN include the anterior insula and dorsal anterior cingulate cortex. Growth curve analyses will be conducted within a linear mixed-effects modelling framework to assess changes in resting-state functional connectivity over time. A linear mixed-effects model will be used to examine both within-subject changes over time (Time 1 vs. Time 2) and between-group differences (experimental group vs. CAN-BIND healthy controls).

## 3. Discussion

As consumer demand for alternative treatments for ADs during the postpartum period increases, this intervention protocol may not only be preferred by some but necessary for others, as medication is not a viable option for many mothers/BPs. This protocol may offer a novel, scalable, inexpensive, and evidence-based non-psychopharmacologic treatment alternative for mothers/BPs experiencing ADs, or a complementary form of treatment for those taking medication but still experiencing clinical levels of anxiety. This intervention is 4 weeks in length, intentionally designed to be shorter than the standard 8-week mindfulness-based programs. While traditional interventions are typically longer, several brief mindfulness protocols have demonstrated effectiveness in reducing symptoms of anxiety and stress. The shorter duration was selected to better accommodate the needs of postpartum mothers, who often face barriers such as limited time, childcare demands, and challenges in access to mental health care during the postpartum period.

While the primary intention of this mindfulness protocol is to mitigate postpartum anxiety and foster mindfulness, its focus on both somatic and psychological symptoms also positions it to yield improvements in emotion regulation and interoception. We expect to observe changes in resting-state functional connectivity, specifically decreased connectivity within the DMN, namely, medial prefrontal cortex–posterior cingulate cortex connectivity (associated with reduced anxiety and improved emotion regulation), and increased connectivity within the SN, particularly anterior insula–dorsal anterior cingulate cortex connectivity (associated with enhanced interoceptive awareness). We expect to observe an attenuation or normalization of DMN connectivity at rest, including medial prefrontal cortex–posterior cingulate cortex coupling, interpreted within a regulation-based framework (rather than uniform suppression). SN analyses are also intended to be exploratory and hypothesis-generating, as interoceptive awareness may be reflected not only in within-network salience connectivity but also in altered interactions between the anterior insula and other large-scale networks (e.g., the DMN).

This will be one of the first studies to supplement self-reported clinical data with neuroimaging in the context of ADs during the postpartum period, providing a more comprehensive understanding of the neural mechanisms underlying mindfulness-based interventions and symptom improvement. It will also likely provide the basis for a larger randomized controlled trial to establish the efficacy of this mindfulness-based protocol in treating postpartum anxiety and its associated symptoms. Given the combined negative impact of ADs in the postpartum period on both the mother/BP and infant, alongside robust evidence supporting the clinical effectiveness of mindfulness for anxiety and the brain’s neuroplasticity potential, we have a unique opportunity to improve health outcomes for mothers/BPs in Canada and beyond.

## Figures and Tables

**Table 1:** Session-by-Session Content of the Mindfulness-Based Group Intervention.

Session 1: Introduction to Anxiety and Mindfulness	- Orientation to group and treatment - Psychoeducation on postpartum anxiety and associated risk factors - Introduction to mindfulness: definition and benefits, awareness vs. automatic pilot, decentering - Formal vs. informal practices - In-group practice: raisin exercise, mindfulness of the breath (i.e., 3 min breathing space), Body scan meditation - Establishing a home practice and goal setting - Formal home practice: body scan meditation, 3 min breathing space - Additional informal home practice: mindfully feeding baby
Session 2: Becoming an Observer of an Anxious Mind, Body, and Experience	- Formal practice (body scan) - Check-in and home practice review - Awareness of thoughts, emotions, and bodily sensations; practicing curiosity, openness, and nonjudgmental awareness - Mindfulness of baby practices (e.g., bath-time, feeding-time, nap-time) - Introducing a pleasant and unpleasant experiences calendar - In-group practice: mindful sitting and mindful movement - Formal home practice: mindful sitting and mindful movement - Additional informal home practice: mindful walking with baby
Session 3: Changing Your Relationship with Anxious Thoughts and Physical Experiences	- Formal practice (mindful movement) - Check-in and home practice review - The CBT component of MBCT - Understanding the difference between helpful and unhelpful interpretations of anxious thoughts and feelings - Acceptance of difficult emotions and life changes, self-compassion - In-group practice: loving-kindness meditation, 3 min breathing space - Formal home practice: loving-kindness meditation, 3 min breathing space - Additional informal home practice: mindful bath-time with baby, mindful nap-time with baby
Session 4: Mindfulness as a Way of Living	- Formal practice (loving-kindness meditation) - Check-in and home practice review - Review of all content and practices introduced in the course - In-group formal extended mindful practice (40 min) - Reasons for continuing practice, and developing a personalized mindfulness practice goal/plan beyond the treatment group

## Data Availability

Data sharing is not applicable to this article, as no datasets were generated or analyzed for this article. Data sharing plans for future analysis will be pre-specified.

## Abbreviations

The following abbreviations are used in this manuscript:

ADs	Anxiety disorders
BPs	Birthing parents
ER	Emotion regulation
TAU	Treatment as usual
DMN	Default mode network
SN	Salience network
MBIs	Mindfulness-based interventions
CEN	Central executive network
DART	Diagnostic Assessment Research Tool
WHCC	Women’s Health Concerns Clinic
IRC	Imaging Research Center
CAN-BIND	Canadian Biomarker Integration Network in Depression
REDCap	Research Electronic Data Capture
STICSA	State-Trait Inventory for Cognitive and Somatic Anxiety
GAD-7	Generalized Anxiety Disorder 7
PSWQ	Penn State Worry Questionnaire
DERS	Difficulties in Emotion Regulation Scale
MAIA-2	Multidimensional Assessment of Interoceptive Awareness 2
ISAQ	Interoceptive Sensitivity and Attention Questionnaire
FFMQ	Five-Facet Mindfulness Questionnaire Short Form
EPDS	Edinburgh Postnatal Depression Scale
MDD	Major Depressive Disorder
PSS	Perceived Stress Scale
SPS	Social Provisions Scale
PBQ	Postpartum Bonding Questionnaire
DAS	Dyadic Adjustment Scale
WHOQoL	World Health Organization Quality of Life Scale
ANOVAs	analysis of variance
